# The Uridine diphosphate (UDP)-glycosyltransferases (UGTs) superfamily: the role in tumor cell metabolism

**DOI:** 10.3389/fonc.2022.1088458

**Published:** 2023-01-19

**Authors:** Wenyu Liu, Jing Li, Rui Zhao, Yao Lu, Panpan Huang

**Affiliations:** School of Basic Medicine, Gannan Medical University, Ganzhou, Jiangxi, China

**Keywords:** UDP-glycosyltransferase, tumor, lipid metabolism, drug metabolism, hormone metabolism

## Abstract

UDP-glycosyltransferases (UGTs), important enzymes in biotransformation, control the levels and distribution of numerous endogenous signaling molecules and the metabolism of a wide range of endogenous and exogenous chemicals. The UGT superfamily in mammals consists of the UGT1, UGT2, UGT3, and UGT8 families. UGTs are rate-limiting enzymes in the glucuronate pathway, and in tumors, they are either overexpressed or underexpressed. Alterations in their metabolism can affect gluconeogenesis and lipid metabolism pathways, leading to alterations in tumor cell metabolism, which affect cancer development and prognosis. Glucuronidation is the most common mammalian conjugation pathway. Most of its reactions are mainly catalyzed by UGT1A, UGT2A and UGT2B. The body excretes UGT-bound small lipophilic molecules through the bile, urine, or feces. UGTs conjugate a variety of tiny lipophilic molecules to sugars, such as galactose, xylose, acetylglucosamine, glucuronic acid, and glucose, thereby inactivating and making water-soluble substrates, such as carcinogens, medicines, steroids, lipids, fatty acids, and bile acids. This review summarizes the roles of members of the four UGT enzyme families in tumor function, metabolism, and multiple regulatory mechanisms, and its Inhibitors and inducers. The function of UGTs in lipid metabolism, drug metabolism, and hormone metabolism in tumor cells are among the most important topics covered.

## 1 Introduction

UDP-glycosyltransferases (UGTs) are a superfamily of enzymes found in animals, plants, fungi, and germs that catalyze the covalent addition of sugars from nucleotide UDP sugar donors to functional groups on a variety of lipophilic compounds. There are 22 UGTs in humans. UGTs found in the endoplasmic reticulum membrane catalyzes the attachment of the hemiacetal hydrogen bond of glucuronic acid (UDPGA) to a range of compounds containing functional groups, such as hydroxy, carboxylic, amino, and sulfhydryl groups to create esters or glycosides. Glucuronides are excreted *via* the bile and urine. The metabolism of many drugs relies on this pathway, as does the excretion of substances, such as endogenous steroids, thyroid hormones, and bilirubin generated from the degradation of heme in the human body (1, 2). To demonstrate the role of glucuronidation in cancer, many reviews have summarized UGTs genetic variants and their risk assessment in cancer, illustrating the effects of UGTs in exogenous carcinogen detoxification and endogenous tumor-promoting factor inactivation (3–5). Reviews of the relationship between UGTs and cancer progression, and that between UGTs and primary or acquired treatment resistance have also been published (6). This review focuses on the function of UGTs in cancer metabolism.

Human UGTs are expressed in a wide range of organs and tissues, but most isoforms are prominent in the liver, kidney and intestine, reflecting their role in detoxification. Tumor cells can meet the high demand for their own growth and survival by promoting the biotransformation of small molecule metabolites. The most common function of UGTs in cancer is the metabolic inactivation of chemotherapeutic agents (7). By conjugating glucuronic acid to lipophilic drugs, UGTs weaken the biological activity of these drugs and increases their water solubility, driving these agents to be eliminated in bile, urine and feces (6). Increasingly, studies have shown that the upregulation and inactivation of UGTs in cancer progression have a significant impact on tumor development as well as prognosis (4, 5, 8). UGTs genes are upregulated in tissues associated with drug metabolism, such as cancers of the liver, kidney, intestine, and pancreas, such as UGT1A6, UGT1A9, UGT1A10, UGT2A3, UGT2B7, and UGT8, and they are significantly associated with increased overall survival in cancer (9). High expression of UGT2B17 in chronic lymphocytic leukemia (CLL) leads to poorer prognosis in CLL, partly because upregulated UGT2B17 glucuronidates anti-leukemic drugs (e.g., fludarabine) in CLL cells, leading to their local inactivation and enhancing their drug resistance (10, 11). It follows that UGTs differentially expressed in tumors can be used as biomarkers or therapeutic targets for cancer prognosis.

Metabolic enzymes are involved in carcinogenesis and metastasis and can be exploited as targets for cancer detection and treatment. Metabolic reprogramming has a significant role in tumorigenesis (12). Metabolic changes in tumors are associated with dysregulation of the activity of intermediate enzymes in metabolic pathways (13). UGTs are rate-limiting enzymes in the glucuronide pathway. Because of the complex and interconnected metabolic networks, changes in the activity of UGTs can affect many metabolic pathways and thus influence tumor development. UGTs have effects on glucose and lipid metabolism in tumor cells in addition to the biotransformation of small molecule metabolites. This article not only describes the role of UGTs in tumors, but also elucidates the new role of UGT in metabolism, including glucose, lipid, drug, and hormone metabolism, providing new research directions for the role of UGTs in tumor metabolism alterations.

The expression and enzymatic activity of UGTs have been reported to be regulated by multiple mechanisms and influenced by a variety of factors. The regulatory mechanisms include epigenetic modifications (e.g. DNA methylation and histone modifications), transcriptional regulation, post-transcriptional regulation (miRNA), and post-translational regulation (e.g., structural and functional modifications, and protein-protein interactions). Methylation regulates UGTs expression in some cases, for example in colon cells, where methylation of the transcription factor HNF1A has a negative regulatory effect on UGT1A1 (14, 15). Histone modifications also regulate the expression of UGTs and also synergize with DNA methylation to regulate the expression of UGT1A1 (16). The promoters upstream of UGTs as well as enhancers comprise the transcription factor conjugating sites that induce and regulate UGTs expression, and the regulation of UGTs by transcription factors varies in different tissues (17). miRNAs can act directly on the mRNA of UGTs to regulate their expression, or indirectly by repressing UGTs transcription factors (18–20). Post-translational N-linked glycosylation and phosphorylation of UGTs and their interactions with different proteins have important effects on their activity (21–23). Therefore, this review contains an in-depth study and summary of its regulatory network and discusses the regulatory relationships of the above multiple mechanisms on UGTs for future studies.

## 2 Roles of UGTs in tumors

Studies have shown that UGTs expression profiles in tumor patients are highly individual and intra-individual specific, and that its upregulation and downregulation correlate significantly with the overall survival of some patients (9). In this review, we summarize the relationship between UGTs expression and breast cancer, lung cancer, liver cancer and prostate cancer.

**Figure 1:**
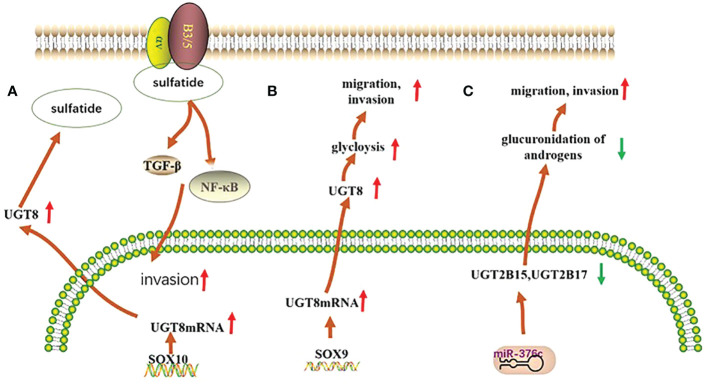
Mechanisms of UGTs role in three types of cancer. **(A)** In basal breast cancer, UGT8 is regulated by SOX10, which promotes the expression of sulfatide and activates the expression of αVβ5 signaling, thereby enhancing the malignancy of basal breast cancer cells; **(B)** In pulmonary non-small cell lung cancer, UGT8 is regulated by the transcription factor SOX9, which affects the glycolytic process in pulmonary non-small cell lung cancer and plays an important role in maintaining the malignancy of pulmonary non-small cell lung cancer and in poor patient prognosis; **(C)** In prostate cancer, the expression of both UGT2B15 and UGT2B17 was negatively regulated by miR-376c, with reduced expression leading to their diminished glucuronidation capacity and consequently to increased tumor malignancy.

UGT family members play an important role in the development of breast cancer. The UGT1A6 gene polymorphism is associated with breast carcinogenesis in the European population, and people with the UGT1A6-19-GC genotype have an increased risk of developing breast cancer (24). Furthermore, the UGT1A8 polymorphism is associated with breast cancer and leads to an increased risk of breast cancer cell malignancy (25). Meanwhile, existing studies have shown that the expression of UGT2B28 has an impact on the metabolic changes of steroid hormones in breast cancer (26). In basal breast cancer, UGT8 enhances the malignancy of basal breast cancer cells. High UGT8 expression is closely related to the tumor grade and size in patients with basal breast cancer, and plays an important role in poor patient prognosis (27) (**Figure 1A**).

Polymorphisms in the UGT1A6 gene in lung tissue and qualitative or quantitative alterations in humans may increase the likelihood of lung cancer in the population (28). High expression of UGT8 in lung cancer tissues can maintain the malignancy of these tissues and is closely associated with drug resistance and tumor metastasis in patients, leading to poor patient prognosis (29, 30) (**Figure 1B**).

UGT1A7 can alter an individual’s susceptibility to cancer by decreasing the body’s detoxification capacity (31). High expression of UGT1A7 in different populations is also strongly associated with increased risk of liver cancer (32). Hepatocellular carcinoma cells can also regulate the expression of UGT family members. In hepatocellular carcinoma, UGT2B4 expression is negatively regulated by miR-135a and miR-410 (33). It was also confirmed that UGT variants were associated with the age of onset, recurrence, distant migration and death in patients with liver cancer (34).

UGT2B15 promotes lymph node metastasis in prostate cancer. Its hypermethylation increases the risk of prostate cancer, and its gene polymorphism is strongly associated with the development of prostate cancer (35, 36). Furthermore, low expression of UGT2B17 further promotes the development of prostate cancer (37). Meanwhile, both polymorphisms are negatively regulated by miR-376c (36)(**Figure 1C**).

The literature indicates that UGTs are important players in tumorigenesis. To investigate whether the UGTs mentioned above also play a role in other cancers, we used bioinformatics analysis to initially explore whether they are of potential research value (GEPIA: http://gepia.cancer-pku.cn/detail.php, SangerBox: http://vip.sangerbox.com/login.html). Elevated expression of UGT1A6 in kidney, liver, and lung cancers compared with normal tissues has different prognostic implications for patients with different cancers. In kidney and liver cancers, the higher the expression of UGT1A6, the shorter the overall survival of patients; while in lung cancer, the higher the expression of UGT1A6, the better the prognosis of patients. This tentatively suggests that UGT1A6 may play an oncogenic role in kidney and liver cancers, while the effect of UGT1A6 on cancer progression in lung cancer needs to be further explored. UGT1A8 expression was elevated in hepatocellular carcinoma, lung cancer, and head and neck squamous cell carcinoma compared with normal tissue, but its expression was not significantly associated with patient prognosis. In adrenocortical carcinoma, UGT1A8 expression was increased, whereas its low expression was associated with poor patient prognosis. UGT8 expression was elevated in colon cancer, esophageal cancer, glioblastoma, low-grade glioma of the brain and gastric cancer compared with normal tissue, and higher expression of UGT8 in colon cancer, esophageal cancer, and gastric cancer was positively associated with good patient prognosis. Furthermore, UGT1A7 expression was elevated in lung cancer and positively correlated with good patient prognosis, while UGT2A3 expression was elevated in kidney cancer compared to normal tissue and positively associated with good patient prognosis. UGT2B15 was highly expressed in hepatocellular carcinoma compared to normal tissues and positively correlated with good patient prognosis. The high expression of UGT1A8, UGT8, UGT1A7, UGT2A3, and UGT2B15 in these cancers and its relation to good patient prognosis is very interesting and deserves further investigation. Hence, increased or decreased expression of UGTs may have an impact on tumor progression. Identifying the key point where UGTs affect tumor progression and blocking it will provide new options for clinical tumor treatment, improvement of patient prognosis, and increasing the survival rate (**Table 1**).

**Table 1:** Relationship between UGTs expression and cancer.

UGTs expression	Types of cancer	The effect of UGTs on cancer	Reference
UGT1A6-19-gc	breast cancer	People with UGT1A6-19-gc have an increased risk of breast cancer.	(24)
UGT1A6 (high)	kidney cancer and liver cancer	Overall survival was shortened.	
UGT1A6	lung cancer	Changes in its gene polymorphism and expression levels increase the likelihood of developing lung cancer.	(28)
UGT1A8 (high)	breast cancer	Breast cancel cells show increased malignancy.	(25)
UGT1A8 (low)	adrenocortical carcinoma	The patients will have a poor prognosis.	(38)
UGT8 (high)	breast cancer	Basal breast cancer cells showed increased malignancy.	
UGT8 (high)	colon cancer, gastric cancer and esophageal cancer	The patients will have a better prognosis.	
UGT1A7*1, UGT1A7*2	liver cancer	Is associated with an increased risk of liver cancer in patients.	(32)
UGT1A7(high)	lung cancer	The patients will have a better prognosis.	
UGT2A3 (high)	kidney cancer	The patients will have a better prognosis.	
UGT2B15 (high)	prostate cancer	Promote lymph node metastasis.	(35)
UGT2B15 (high)	liver cancer	The patients will have a better prognosis.	
UGT2B17(low)	prostate cancer	Promoting the development of prostate cancer	(37)

## 3 Roles of UGTs in the regulation of tumor metabolism

The metabolic networks in the human body are interconnected to achieve homeostasis due to the interaction of metabolic signals. In the process of cancer development, any dysregulation of metabolites will lead to the disruption of the metabolic network, which leads to cancer development and malignant metastasis. The glucuronate pathway’s rate-limiting enzyme belongs to the UGTs. Alterations in its metabolism can affect gluconeogenesis and even lipid metabolism pathways, leading to altered metabolism in tumor cells, which affects the development and prognosis of cancer.

### 3.1 Roles of UGTs in tumors related to glucose metabolism

**Figure 2:**
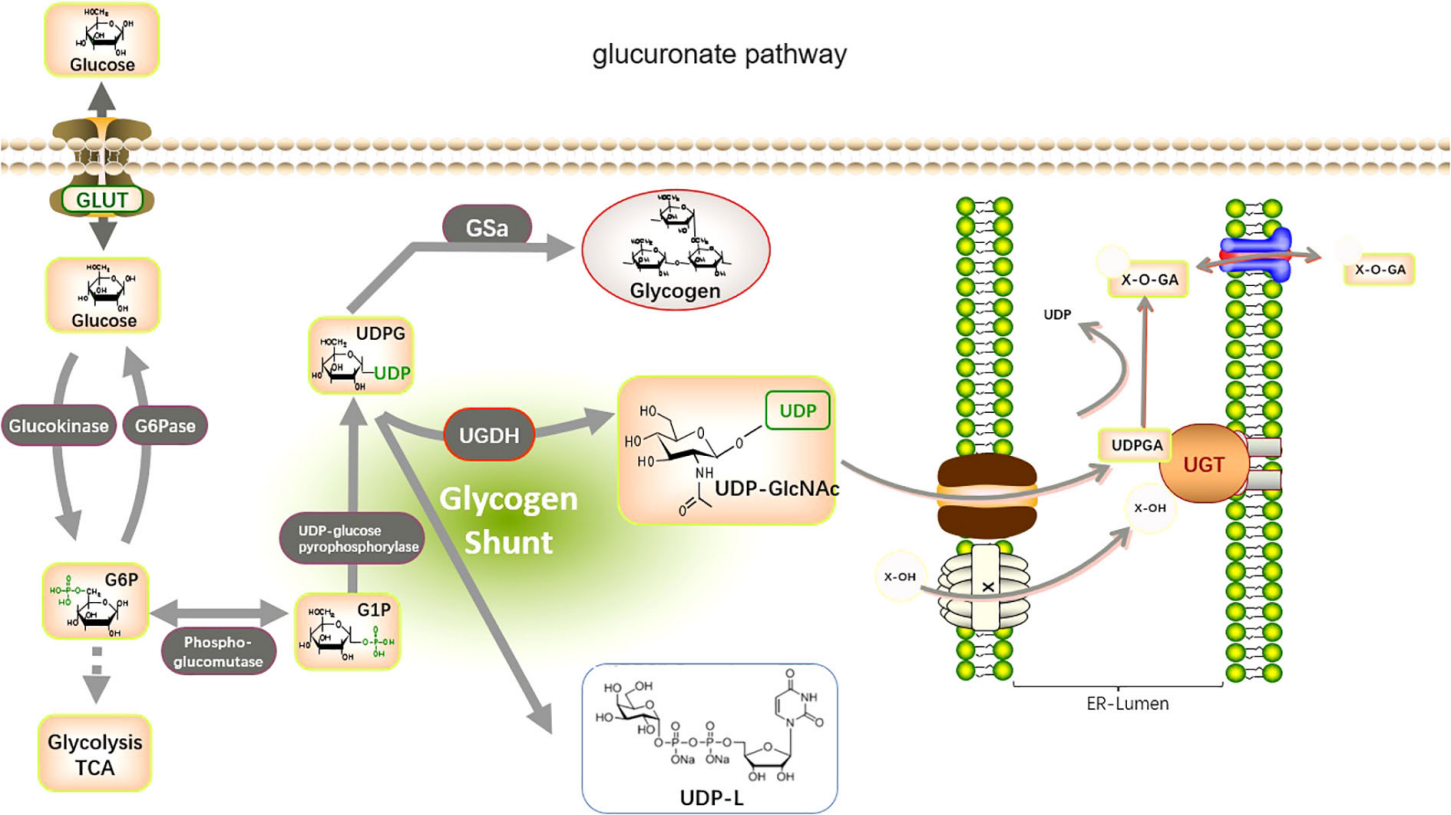
Metabolic pathways involved in glucuronidation by UGTs. The glucuronate pathway is a branched pathway of sugar metabolism with a small flux. The first stage of the glucuronate pathway is the isomerization of the glycolytic intermediate, glucose 6-phosphate, to produce glucose 1-phosphate. This is then reacted with UTP to produce UDP-glucose. The catalytic enzymes are phosphoglucomutase (PGM) and UDP-glucose pyrophosphorylase (UGP). UDP-glucose is an important branch point of sugar metabolism. It can be used for the synthesis of UDP-glucuronide, UDP-galactose, and also as an entry point for polysaccharide synthesis. UDP-glucose is catalyzed by UDP-glucose dehydrogenase (UGDH) to produce UDP-glucuronide. This reaction involves two successive oxidation steps that oxidize the terminal hydroxymethyl group of glucose to a carboxyl group while generating two NADH. UDP-glucuronide is an important substance used by the liver for detoxification and can react with many fat-soluble substances (**Glucuronidation**). this reaction is catalyzed by UDP-glucuronyl transferase (UGTs) in the endoplasmic reticulum membrane, and the hemiacetal hydroxyl group of glucuronide can be combined with a variety of substances containing functional groups such as hydroxyl, carboxyl, amino, and sulfhydryl groups to produce esters or glycosides, which are excreted from the bile and urine.

The UGTs are involved in the metabolism and elimination of thousands of both exogenous and endogenous human hydrophilic drugs and substances. UGTs conjugate a variety of tiny lipophilic compounds to sugars, such as glucuronide, galactose, glycosyl, or galacto (39–42), with substrates, such as cancer-causing substances, medications, corticosteroids, triglycerides, fatty acid oxidation, and bile salts (43). UGT1A, UGT2A, and UGT2B are primarily responsible for this reaction, which is known as glucuronidation. UGT covalently conjugates with other substrates *via* glucuronic acid provided by UDP-sugars. These compounds are then removed from the body through bile, urinary, and fecal matter (44) (**Figure 2**). UGT8 is a UGT family member that catalyzes the transfer of galactose from UDP galactose to ceramide, a crucial step in the synthesis of brain sphingolipids. In contrast, UGT3A1 and UGT3A2 use UDP N-acetylglucosamine and UDP glucose and UDP xylose, respectively, as sugar donors to conjugate substrates. Furthermore, it has been demonstrated that UGT8 is also capable of conjugating bile acids and bile acid analogs that are similar to drugs, such as galactosylation, and that UGT8 conjugates bile acids around 60 times more effectively than ceramide (3, 42).

In addition to the function of UGTs in glucuronidation, UGTs may interact with other metabolic enzymes thereby affecting multiple metabolic pathways in tumor biology and have an impact on the alteration of the tumor phenotype (6). It has been shown that a transcription factor promotes the high expression of UGT8 in non-small cell lung cancer (NSCLC) and that UGT8 upregulation maintains the malignancy of NSCLC by enhancing glycolysis (29). UGT1 interact with the rate-limiting enzyme of glycolysis pyruvate kinase (PKM2) in colon cancer cells contributing to cancer cell metabolism and tumor growth (45). Changes in the enzymatic activity of UGTs may have an impact on the energy production of “aerobic glycolysis,” which is essential for the growth of cancer cells. The glucuronidation activity of UGTs may also affect UDP glucose metabolic pathways, including UDP glucose, UDPGA, and UDP xylose, which are derived from glucose-6-phosphate, an intermediate product of glycolysis. According to a theory formed in the context of prostate cancer advancement, alterations in the enzymatic activity of UGTs may also impact how well their byproducts perform, such as the many functions of UDPGA in glucuronidation or the production of UDP xylan and proteoglycan. UDPGA appears to be preferred in the synthesis of proteoglycans (e.g., NOTCH1) in androgen-independent cells, possibly to prevent inactivation of intracellular testosterone depots, according to studies in prostatic cancer cell types (46). Based on this, alterations in the UGT enzyme family can affect not only their own functions but also the functions of other enzymes of the glucuronate pathway or other metabolites, thereby affecting tumor development. In turn, alterations in the glucuronate pathway may affect the entire gluconeogenic pathway. As the center of the metabolic network, changes in glucose metabolism are inextricably linked to other metabolic pathways, such as lipid metabolism and nucleotide metabolism. Therefore, UGTs affect tumors by regulating metabolism, providing a new direction for future research on the metabolic mechanisms of tumors.

### 3.2 Roles of UGTs in tumors related to lipid metabolism

UGTs do not only glucuronidate estrogens, androgens and bile acids, which are common cholesterol-derived molecules. They also react with some other fatty acid derivatives, altering their biological activity and degrading many low molecular weight endogenous molecules that alter some endogenous substrates with oncogenic functions, such as vitamin A, leukotriene B4, prostaglandins, and arachidonic acid precursors (47–50). For example, it has been shown that UGT2B17 can glucuronidate prostaglandin E2 (PGE2) thereby affecting the frontal proliferation and migratory capacity of leukemia cells (47).

**Figure 3:**
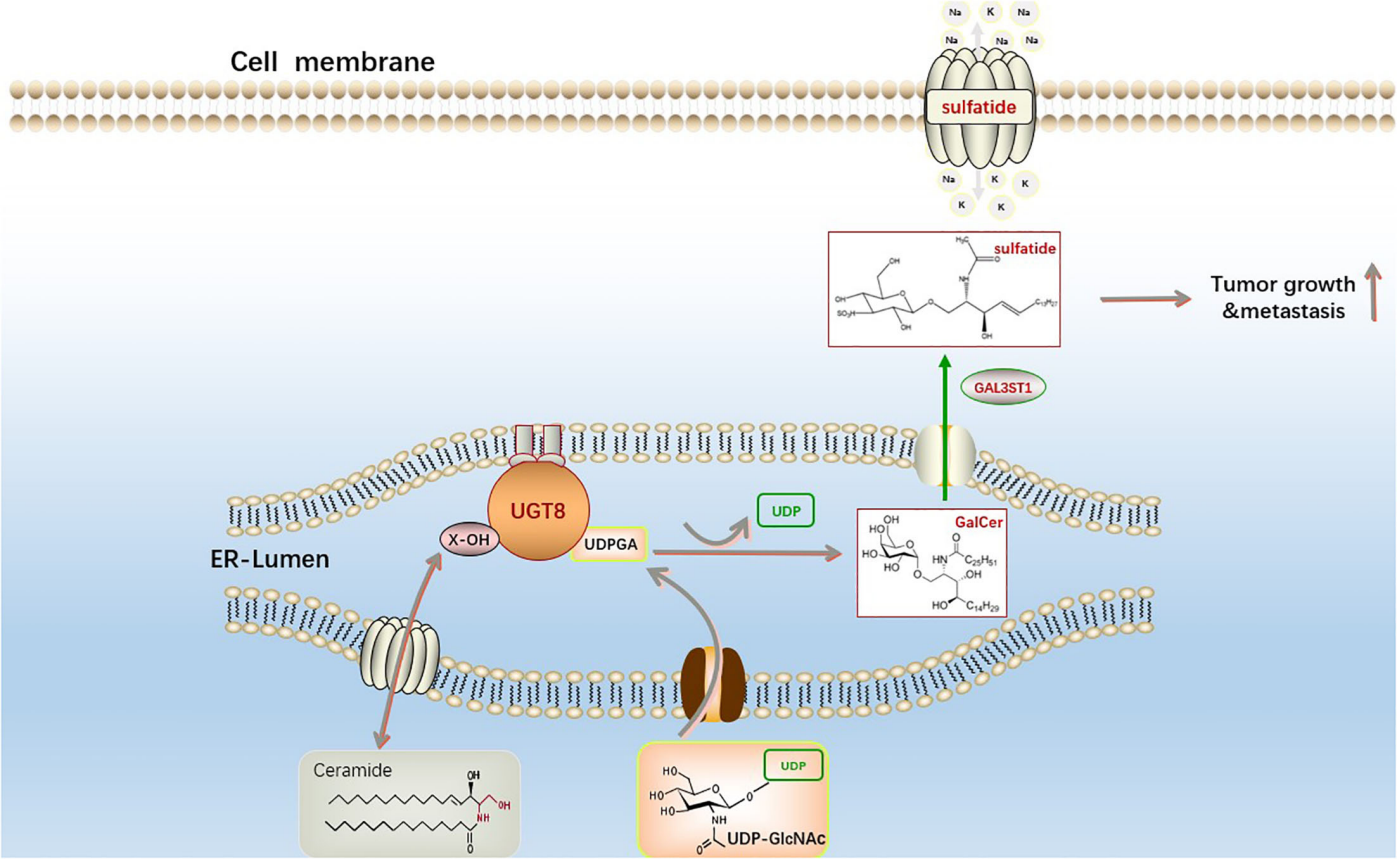
The synthesis pathway of sphingosine sulfatide. The first step is catalyzed by UGT8 on the endoplasmic reticulum to generate GalCer and uridine diphosphate (UDP) from ceramide and uridine diphosphate galactose (UDP-galactose). The second galactoceramide transferase (GAL3ST1) in this reaction converts galactoceramide to Sulfatide *via* a sulfonation reaction. Sphingosine sulfatide on the cell membrane is involved in the regulation of proliferation, differentiation, apoptosis and senescence of cancer cells.

Changes in lipid metabolism can have a profound clinical effect on breast cancer since they can increase the spread and recurrence of the disease (51). Ceramides can be galactosylated by ceramide galactosyltransferase (UGT8) to form galactosyl ceramides (GalCer), which are then converted to sulfate by GalCer sulfotransferase (GST). UGT8 is a crucial enzyme in the metabolism of sulfatidylcholine and is abundantly expressed in the brain and nervous system. Additionally, a recent study discovered that GalCer was a crucial glycosphingolipid for both the central and peripheral nervous systems, and that UGT8 deficiency caused significant neurological impairment (52). UGT8 is a key enzyme in the synthesis of sulfatide, a sphingolipid widely found in eukaryotic cells. Sulfatide is a type of lipid that plays an important part in the development of numerous diseases, such as disorders of the neurological system, cardiovascular disease, diabetes, immunological disorders, cancers (53). Sox10 acts as a transcription factor to promote the expression of UGT8, which significantly promotes ceramide metabolism leading to sulfatide formation and thus increases the binding of integrin αVβ5 to activate the TGF-β and NF-κB pathways in basal-like breast cancer to encourage its capacity to spread and migrate (38) (**Figure 3**). Many malignancies exhibit reprogramming of lysosomal metabolism. It has been discovered that UGT8 is linked with tumor progression and that elevated UGT8 levels may be crucial for the emergence of lung metastases (54). UGT8 has also been found to be substantially prevalent in cervical and oropharynx malignancies through analysis of clinical samples (55, 56). Ceramide functions differently in different cancer cells as a second messenger of intracellular signaling, and there is clear evidence that one of the causes of drug resistance is ceramide glycosylation (57–59). These investigations demonstrate that UGT8 can maintain intracellular ceramide levels, raise glycosphingolipid levels, control drug transport, lessen cell death, and combat drug resistance. Breast cancer cells with high UGT8 expression exhibit sensitive to apoptosis caused by adriamycin. According to these studies, increasing UGT8 expression can convert ceramide to GalCer and reduce ceramide-induced apoptosis (60). The survival of cancer cells may highly depend on this mechanism. By blocking the glucuronidation of ceramide, or finding effective inhibitors of UGTs, new ideas have been generated for studying the development and prognosis of tumors.

### 3.3 Role of UGTs in the metabolism of antitumor drugs

UGT family members, which are highly expressed in tissues related to drug metabolism, promote drug metabolism *in vivo* through glucuronidation and play a key role in the metabolism of some antitumor drugs, leading to improved drug function and reduced drug toxicity. For example, the drug irinotecan, used to treat small cell lung cancer, has serious drug toxicity. It is converted to the active metabolite SN-38 *via* carboxylesterase, and UGT1A1 mediates the conversion of SN-38 to an inactive compound by binding to SN-38G, facilitating its excretion from the body (61). An evaluation and analysis of a large body of literature revealed a high occurrence of severe irinotecan-induced toxicity in pure carriers of the UGT1A1 mutant UGT1A1*28 or UGT1A1*6; hence, these UGT1A1 mutations were predictive of irinotecan-related toxicity (62). UGT1A6 is involved in the oxidative metabolism of benzo(a)pyrene (BaP) and BaP quinone exerting a detoxifying effect, and the combination of UGT1A6 and cytochromes (CYPs) can further enhance its detoxifying effect and reduce the toxic effect of carcinogens (63). Regarding polycyclic aromatic hydrocarbon (PAH) carcinogens, UGT1A4 is the main active enzyme in the glucuronidation of the nicotine secondary metabolite trans-30-hydroxycotinine (64). It has been shown that resveratrol, as an anticancer drug for breast cancer, can inhibit the development of breast cancer cells by upregulating the expression of NRF2 and UGT1A9, promoting the metabolism of estrogen in the body, and inhibiting the cell damage caused by toxic metabolites produced by estrogen (65). UGT2A1 counteracts the activity of simple and complex PAHs, and has a mitigating effect on lung cancer caused by long-term exposure to PAHs (66). UGT2A2 is a splice variant of UTG2A1, which is expressed mainly in the nasal mucosa and plays an important role in the local detoxification of carcinogenic monohydroxy PAH metabolites (67, 68). Unlike UGT2A1, UGT2A2 has no glucuronidation activity toward TSNAs, HCA, or nicotine and does not possess N-glucuronidation ability (69). UGT2A3 and UGT3A1 can only detoxify simple PAHs because of their weak activity in the human body (69, 70).

In addition to the beneficial role of drug detoxification, UGT has a detrimental effect on the human body. When UGT1A9 interacts with bisphenols, it leads to intracellular calcium overload, which induces mitochondrial stress, leading to dysregulation of mitochondrial homeostasis, and promoting bisphenol-induced cell death (71). UGT1A10 has also been associated with glucuronidation of the acridone derivatives C-1305 and C-1311 antitumor drugs, significantly increasing the cytotoxicity of C-1305, enhancing its pro-apoptotic properties in HCT116 cells and leading to inactivation of exogenous substances (72). Bitter almond phenol, which has anticancer effects, can form three metabolites (M1-M3) after glucuronidation. UGT1A10 mainly catalyzes the formation of M2, which can affect the clearance of its metabolites and affect their bioavailability (73). The UGT2B11 mRNA affects the IC50, EC50, and AUC of anti-prostate cancer drugs and confers resistance to cisplatin-based drugs (74–76). UGT2B17 is involved in the glucuronidation of exemestane, an aromatase inhibitor against breast cancer, and its copy number variation leads to individual differences in drug metabolism (77, 78). It is also involved in the inactivation of the anti-leukemia drugs fludarabine and ibrutinib, leading to the development of resistance to these drugs in patients (10, 79). Unlike other UGTs, UGT1A7 has a dual role: it inhibits irinotecan and erlotinib for rectal and small cell lung cancers and is also involved in the inactivation of various carcinogens including hydroxybenzopyrene metabolites. In contrast, it can promote the action of ketoconazole so that it can be used for infections caused after chemotherapy (80) (**Table 2**). Thus, members of the UGTs mainly function in the metabolism of drugs and carcinogens by participating in their glucuronidation. However, some members are involved in the metabolism of these substances by other means. They have both beneficial and detrimental effects on the organism, which are closely related to the polymorphism of the UGTs gene and the enzymes that act in concert with it. Elevated UGT2B11 expression during the treatment of prostate cancer with cisplatin-based drugs may indicate that the body has developed resistance to these drugs and that androgen deprivation therapy or immunotherapy may be used to treat the prostate cancer (81, 82). In addition, after treating breast cancer with exemestane, the polymorphism of the UGT2B17 gene leads to individual differences in the drug and the upregulation of UGT2B17 expression in some patients may be related to the development of drug resistance (78). The treatment of radiotherapy or endocrine therapy, or integrated treatment may be a good alternative therapy (84). Elevated expression of UGT2B17 also occurs in the treatment of leukemia with fludarabine or ibrutinib, which may also suggest the exists of drug resistance (10). Taken all together, Tyrosine kinase inhibitors (TKI) targeting BCR-ABL1 tyrosine kinase, monoclonal antibodies targeting cell surface antigens (CD19, CD20, and CD22), bispecific antibodies, and chimeric antigen receptor (CAR)-T therapy may be good alternative treatments (85).

**Table 2:** Relationship between UGTs expression and adverse drug effect.

UGTs expression	Type of Disease	Targeted drugs	Route of action	Alternative treatment options	Reference
UGT2B11(high)	Prostate Cancer	Cisplatin-based drugs	affect the IC50, EC50, AUC of this class of drugs	Androgen deprivation therapy, immunotherapy	(81, 82)
UGT2B17(high)	Breast Cancer	Exemestane	Affects the glucuronidation of this class of drugs	Radiotherapy, endocrine therapy	(78)
UGT1A9(low)	Breast Cancer	Resveratrol	Resveratrol upregulates the expression of NRF2 and UGT1A9 to promote estrogen metabolism *in vivo*	Radiotherapy, endocrine therapy	(65, 78)
UGT2B17(high)	Leukemia	Fludarabine, Ibrutinib	Involvement in drug inactivation	TKI, monoclonal antibodies targeting cell surface antigens, CAR-T	(10)
UGT1A1(low)	Small cell lung Cancer	Irinotecan	Mediated conversion of SN-38 to an inactive state	Immunotherapy, cisplatin-based drugs	(61, 83)
UGT1A1*28, 1A1*6(high)	Small cell lung Cancer	Irinotecan	Not yet reported	Immunotherapy, cisplatin-based drugs	(62, 83)
UGT1A7(high)	Small cell lung Cancer, Rectal Cancer	Irinotecan	Affects the glucuronidation of drugs	Small cell lung cancer: As above, Rectal Cancer: Neoadjuvant radiotherapy	(32, 80)
UGT1A7(low)	Post-chemotherapy infection	Ketoconazole	Affects the glucuronidation of drugs	Change to other antimicrobials	(32)

* is the allelic variation of UGT gene.

### 3.4 Role of UGTs in hormone metabolism

Members of the UGTs play an important role in hormone metabolism. UGT1A1 plays an important role in estrogen metabolism. It is highly expressed in the uterus and is involved in the elimination of estrogen (61). After menopause, women gain weight and fat content, with a subsequent increase in estrogen sources, which leads to a decrease in bone transformation and an increase in bone loss (61). Analysis of postmenopausal women with osteoporosis revealed that UGT1A1*28 can be used as a marker of bone loss for the timely assessment of bone tissue changes, and that pureton mutations in UGT1A1*28 can reduce the risk of bone loss and osteoporosis in postmenopausal women (25, 61). Excessive accumulation of estrogen and its toxic metabolites can stimulate abnormal proliferation of breast cells, which causes breast cancer, while UGT can react with estrogen, which promotes the metabolism of estrogen and play a certain detoxifying effect (86). *In vivo*, UGT1A7 and UGT1A8 can participate in the glucuronidation of estrogen and promote estrogen metabolism. UGT1A7 can also participate in the glucuronidation of estrogen metabolites catechol estrogen and methoxyestradiol metabolites (65). In breast cancer tissues, polymorphisms in specific UGTs genes regulate the exposure to toxic estrogen metabolites. UGT1A8 expression is regulated by NRF2 and reduced UGT1A8 expression leads to estrogen accumulation in the body and increased cellular damage (25, 65). UGT2B11 plays a catalytic role in the glucuronidation of androgens (74–76). It is also involved in the glucuronidation of steroids and promotes the excretion of toxic substances from target cells (87). UGT2B28 is abundantly expressed in the human liver and kidney and is involved in the metabolism of estradiol and androstenedione. Its altered function can interfere with HBV replication by affecting the metabolism of sex hormones (34, 88). One study showed using multivariate analysis that UGT2B28 gene mutations are closely associated with the development of hepatocellular carcinoma, the ability to metastasize to distant sites, and the age of onset, which may be related to its involvement in HBV replication (34). UGT2B28 also acts as a regulator of steroid hormones and alters testosterone dihydrotestosterone levels. In primary prostate cancer, if androgen expression is elevated, UGT2B28 expression is also elevated (89), therefore, UGT2B28 can be a good predictor of prostate cancer. UGT2B15 and UGT2B17 play an important role in the metabolism of androgens and are involved in the inactivation of testosterone and dihydrotestosterone (36, 90). UGT2B17 expression is negatively regulated by the androgen receptor. In prostate cancer cells, androgens can inhibit UGT2B17 expression by activating the androgen receptor, and glucuronidation leads to increased androgen secretion in the body, which will promote prostate cancer progression, forming a vicious cycle (37).

Members of the UGTs also play an important role in the metabolism of bile acids. UGT2A1 and UGT2B4 are highly responsive to bile glucuronidation (91). UGT2B28 is involved in bile acid metabolism and can explain the correlation between bilirubin levels and high alcohol consumption (89). UGT3A1, in contrast, is involved in the metabolism of ursodeoxycholic acid, a therapeutic drug for patients with cholestatic liver disease, and catalyzes the detoxification of bile and urine by ursodeoxycholic acid, which plays an important function in patients with cholestasis (39). Recently, it has been found that UGT8 also plays an important role in the metabolism and clearance of bile acids, not only in the maintenance of bile acid homeostasis *in vivo* but also in bile acid signaling (42). As a conjugating enzyme in the endocrine system, UGTs play a major role in the metabolism of catecholamine hormones as a conjugating enzyme in the endocrine system and is involved in the development of these hormone-related cancers (92). (**Table 3**)

**Table 3:** Relationship between expression of UGTs and hormone metabolism.

UGTs Expression	Affecting hormones	Specific Mechanisms	Reference
UGT1A1 (high)	Estrogen	Involved in estrogen conjugating and elimination	(61)
UGT1A1^*^28 pure mutation	Estrogen	Reduce the risk of bone loss and osteoporosis in postmenopausal women	(61)
UGT1A7 (high)	Estrogen, Catechol estrogen	Participate in their glucuronidation and promote their metabolism	(65)
UGT1A8 (low)	Estrogen	Causes estrogen build-up in the body and increased cell damage	(65)
UGT2B11 (high)	Androgen	Catalyzing the glucuronidation of androgens	(75)
UGT2B11 (high)	Steroid hormones	Participates in glucuronidation of steroid hormones and promotes the excretion of toxic substances	(87)
UGT2B15 (high)	Androgens	Involving in the inactivation of testosterone and dihydrotestosterone	(36)
UGT2B17 (high)	Androgens	Involving in the inactivation of testosterone and dihydrotestosterone	(36)
UGT2B28 (high)	Estradiol, Androstenediol	Affects sex hormone metabolism, which in turn interferes with HBV replication	(34, 88)
UGT2B28 (high)	Androgen	Altered testosterone dihydrotestosterone levels may predict prostate cancer	(89)
UGT2B28 (high)	Bile acids	Explain the correlation between bilirubin levels and patients after heavy alcohol consumption	(89)
UGT3A1 (high)	Ursodeoxycholic acid	Participates in the metabolism of ursodeoxycholic acid and catalyzes its detoxification	(39)
UGT8 (high)	Bile acids	Maintenance of bile acid homeostasis and signaling	(42)

* is the allelic variation of UGT gene.

## 4 UGTs are regulated by multiple regulatory mechanisms

The UGTs plays an important role in the metabolism and clearance of a wide range of substances, and there is growing evidence that the UGTs plays a functional role in the development of many diseases, particularly in cancer development. Moreover, the UGTs is controlled at multiple levels by a variety of factors, including epigenetic modification, transcriptional regulation, post-transcriptional regulation and post-translational regulation. Therefore, this review provides an in-depth study and summary of their regulatory networks.

### 4.1 Epigenetic modification

UGTs expression is tissue-specific. For example, UGT1A7, UGT1A8, and UGT1A10 are only expressed in the gastrointestinal tract, whereas UGT1A9 is stably expressed in the liver and kidney (93). In addition, the expression of some UGTs isoforms has been shown to be closely associated with DNA methylation (14). The CpG-enriched region near the UGT1A1 promoter is highly methylated in the kidney and hypomethylated in the liver, and DNA methylation is negatively correlated with UGT1A1 expression (14, 15). This could partly explain the tissue-specific expression of UGT1A1. Similarly, the tissue-specific expression of UGT1A10 in the liver and intestine may also be associated with methylation (94).

It has been shown that histone modifications can regulate UGT1A1 expression in the liver. For example, the enrichment of the transcriptional activation marker H3K4me2 in the adult liver is closely associated with high expression of UGT1A1, while the enrichment of the UGT1A1 transcriptional repression marker H3K4me3 is consistent with repression of fetal UGT1A1 expression, both of which are the result of histone modifications (95). By way of comparison, it is reasonable to surmise that histone modifications may synergistically regulate gene expression with DNA methylation, such as histone modifications and DNA methylation synergistically regulating the expression of UGT1A1 in the kidney (16).

Studies show that there are significant gender differences in disease occurrence. The mechanism underlying these phenomena is probably related to differences in the regulation of gene expression, especially that related to sex hormones (96). Some scientists have studied the relationship between estrogen receptor α (ERα) and the sex-specific expression of UGT1As. Studies have shown that ERα binds to the xenobiotic response element (XRE) of UGT1As by recruiting histone deacetylases 1 and 2, thus significantly inhibiting the transcription of *UGT1A* (97). This indicates that chromatin remodeling induced by histone modifications is involved in the sex differential expression of UGT1As.

In summary, studies confirm that histone modifications do play a crucial role in UGTs gene expression and that UGTs expression is clearly regulated by DNA methylation. However, the current understanding of the epigenetic mechanism of UGTs is very limited and further studies are needed to elucidate the association between other isoforms of UGTs and epigenetics.

### 4.2 Transcriptional regulation

The promoter upstream of UGTs, as well as the enhancer, comprise the sites of transcription factor binding that induce and regulate UGTs expression. The regulation of UGTs by transcription factors varies in different tissues, and in the liver UGTs expression is regulated by the transcription factors HNF1 and HNF4, PXR, CAR, PPARα, and the Ah receptor (AhR) (17). It has been shown that HNF1 is able to interact with CAR, PXR, AhR, and GR as a regulator essential for their promotion of UGT1A1 expression, while HNF4 can further reduce UGT1A1 expression *in vivo* by inhibiting the expression of these receptors. AhR is expressed in almost all UGT1 members and in the nucleus by binding to ARTN and promoting HSP90 dissociation from AhR and binding to XRE located in the promoter of its target gene, thereby promoting the expression of UGT family members and thus stimulating their glucuronidation ability. Nrf2 also regulates UGTs expression through binding to ARE. PXR and CAR can be involved in cholesterol metabolism and it has been shown that they are abundantly expressed in the liver as nuclear receptors, and that UGTs are their effective target genes. They can regulate their own activity in a cell cycle-dependent manner, thus affecting the expression of UGT family members. They can also be activated by a variety of anti-lipid drugs, which in turn promote UGTs expression, facilitating drug glucuronidation, reducing drug toxicity, and decreasing damage to the organism (98). GR may be synergistically involved when UGT1A1 is involved in the metabolism of exogenous substances, whereas CAR/PXR regulates UGT1A1 expression and influences the regulation of exogenous responses by UGT1A1 (98, 99). The regulators of UGT expression form a feedback loop with UGTs substrates. For example, UGT1A1 promotes the glucuronidation of bile acids, facilitating their metabolism in the body and reducing bile acid accumulation in the body. Bile acids in the body can also act on the transcription factors PXR, CAR, and AhR of UGTs to stimulate their activity and further promote the expression of UGT. The same feedback loop also occurs between hepatotoxic bile acids, UGT2B4, UGT2B7, and FXP, PPARα; between some eicosanoids, PPARa and UGTs; and between dietary polyphenols, UGTs and Ah receptors, which would provide a new basis for further investigation of the characteristics of UGTs action on substrates. However, the interactions between UGTs transcription factors in the loop are unclear that still need to be further explored (100).

### 4.3 Post-transcriptional regulation – MicroRNA

MiRNAs are endogenous non-coding RNAs consisting of 19-25 nucleotides that regulate gene expression by translational repression or degradation of mRNA through incomplete base pairing with the target mRNA (101). Growing evidence shows that miRNAs play a role in essential cellular functions and, as such, abnormal miRNA regulation is associated with the development and progression of a wide range of diseases.

Studies have confirmed that mir-491-3p binds to the 3’UTR of UGT1A, and that its overexpression can significantly inhibit the mRNA levels of UGT1A1, UGT1A3, and UGT1A6. In contrast, inhibition of mir-491-3p expression leads to an increase in UGT1A mRNA level and activity (18). Similarly, mir-141-3p downregulated the mRNA expression and activity of UGT1A1 and UGT1A6 in LS180 and human hepatocytes (19). This indicates that both mir-491-3p and mir-141-3p are among the factors regulating the expression of UGT1A in the liver. It was also found that miR-216-5p downregulated the expression of UGT2B4, UGT2B10, and UGT2B15, and luciferase assays showed that miR-216b-5p bound to the MRE on the 3’UTR of UGT2B7, UGT2B4, and UGT2B10 (102). Similarly, miR-135a and miR-410 downregulated UGT2B4 expression in HepG2 and Huh-7 cells by binding the 3’UTR, and miR-3664 downregulated UGT2B7 expression by binding the 3’UTR (33).

In addition, miRNAs can also regulate the UGTs expression profile through indirect mechanisms. It was found that the expression level of mir-375 in the low UGT1A activity group was significantly higher than that in the high UGT1A activity group. At the same time, it was found that the binding site of differentially expressed mir-375 was not on the UGT1A 3’UTR, but on the AhR mRNA. This is a transcription factor that has been clearly shown to regulate UGT1As. Similarly, overexpression of mir-137 in LS180 cells decreased the expression of AhR target genes UGT1A1 and UGT1A6. Thus, miR-137 and miR-375 can indirectly downregulate the expression and activity of UGT1A1 by inhibiting the expression of the transcription factor AhR (20).

The expression of miRNAs often differs markedly between healthy individuals and patients, and aberrant regulation of miRNAs is closely associated with disease, further making them a focus of biomarker research (103). UGT1A1 plays a crucial role in the metabolism of ketamine *in vivo*, while mir-548D-5p mainly binds to the 3’UTR of UGT1A1 and inhibits the expression of UGT1A1 mRNA and protein in hepatocytes. Based on this, it was found that in patients with low ketamine treatment efficacy, the low expression of mir-548D-5p led to high UGT1A1 activity and high level of glucuronidation in hepatocytes, thereby accelerating the metabolic clearance of ketamine drugs and reducing the therapeutic effect (104). Similarly, in patients with hepatocellular carcinoma treated with sorafenib after surgery, those with good prognosis had high UGT1A9 expression. Gene monitoring assays confirmed a negative post-transcriptional regulation of UGT1A9 expression by miR-200a/-183, with low levels of miR-200a/-183 suggesting high levels of UGT1A9, thereby increasing sorafenib β-diglyceride formation in HCC and enhancing drug efficacy (105). miRNA regulation of UGT expression and activity also plays an important role in tumor development. Several groups have reported the importance of miR-376c in downregulating UGT2B15 and UGT2B17 in prostate cancer. Luciferase assays showed that miR-367c directly binds to the 3’UTR of UGT2B15 and UGT2B17, and that overexpression of miR-376c downregulated UGT2B15 and UGT2B17, further reducing testosterone and androgen glucuronidation in prostate adenocarcinoma cells in response to elevated residual androgen levels in the organism. Prostate cancer cell proliferation was enhanced (36, 106).

**Figure 4:**
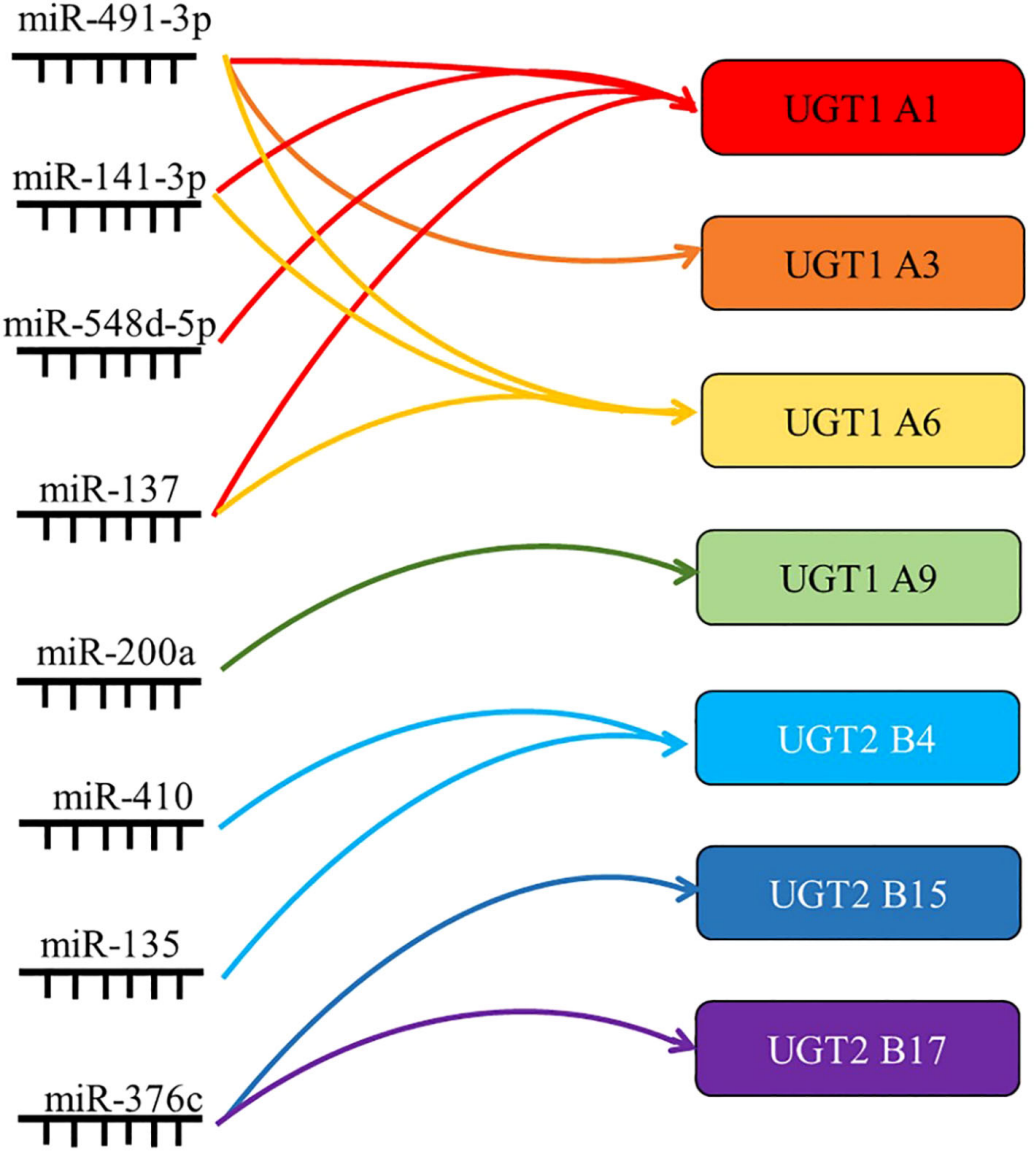
Summary of the targets of miRNAs associated with UGTs. miRNAs regulate gene expression by translational repression or degradation of mRNAs through incomplete base pairing with target mRNAs. By summarizing the direct regulatory role of miRNAs, their indirect regulatory role and their role in drug metabolism and tumor progression, the most important UGTs targets are listed here.

To better understand the post-transcriptional regulation of UGTs, more in-depth and comprehensive studies targeting miRNAs are needed (**Figure 4**).

### 4.4 Post-translational modifications

The only post-translational modifications of UGTs are N-linked glycosylation and phosphorylation (21). The post-translational modifications of UGT1A6, 1A9, 2B7, 2B15, and 2B17 include glycosylation. N-linked glycosylation plays an important role in the correct folding of these proteins and preservation of enzymatic activity and can also affect the interaction of UGTs with other proteins of the endoplasmic reticulum (21, 22). Owens et al. found that the phosphorylation regulation with Protein kinase Cα and Src kinase as the core plays a key role in maintaining the activity levels of UGT1A7, UGT2B7, and UGT2B15. In addition, mutations in the predicted site of PKC and the Src site likewise greatly reduce enzyme activity, together elucidating the kinases and mechanisms involved in UGTs phosphorylation (107, 108). In summary, the complex pattern of glycosylation and phosphorylation regulation in the organism is necessary for homeostasis. The phosphorylation of UGT2B is tyrosine-dependent, and it has been shown that mutations in the phosphorylation sites of UGT1A7, UGT1A10, UGT2B7, and UGT2B15 reduce the activity of these enzymes. The optimal activity of UGTs is maintained by phosphorylation (21, 22).

### 4.5 Involvement in protein–protein interactions

UGTs family proteins can interact with proteins of the same isoform and also with proteins of different isoforms. Interactions between UTG isoforms affect their activity (21, 23). UGT1A1 is able to interact with UGT1A3, UGT1A4, UGT1A6, UGT1A7, UGT1A8, and UGT1A9, and the interaction of UGT1A1, 1A9, and 2B7 affects enzyme activity and also alters their regioselectivity for substrates (22). In patients with hyperbilirubinemia with low UGT1A1 activity, UGT1A1 enhances the metabolism of UGT1A9 for the anticancer drug sorafenib (21, 109). *In vivo* UGT2B7 activity decreases and the metabolism of its specific substrate isoproterenol by UGT1A9 is reduced (21). The activity of UGT1A4, UGT2B4, and UGT2B7 is also affected by the inhibition of UGT1A9 expression (21, 110). The effect of UGT1A4 and UGT1A6 on UGT1A1 activity upon their interaction with UGT1A1 depends on the substrate on which they act (111). The identical isoforms UGT2B4, UGT2B7, and UGT2B17 can also interact. Their interaction mostly affects some of their specific substrates and is influenced by cross splicing and genetic metabolism (112).

**Figure 5:**
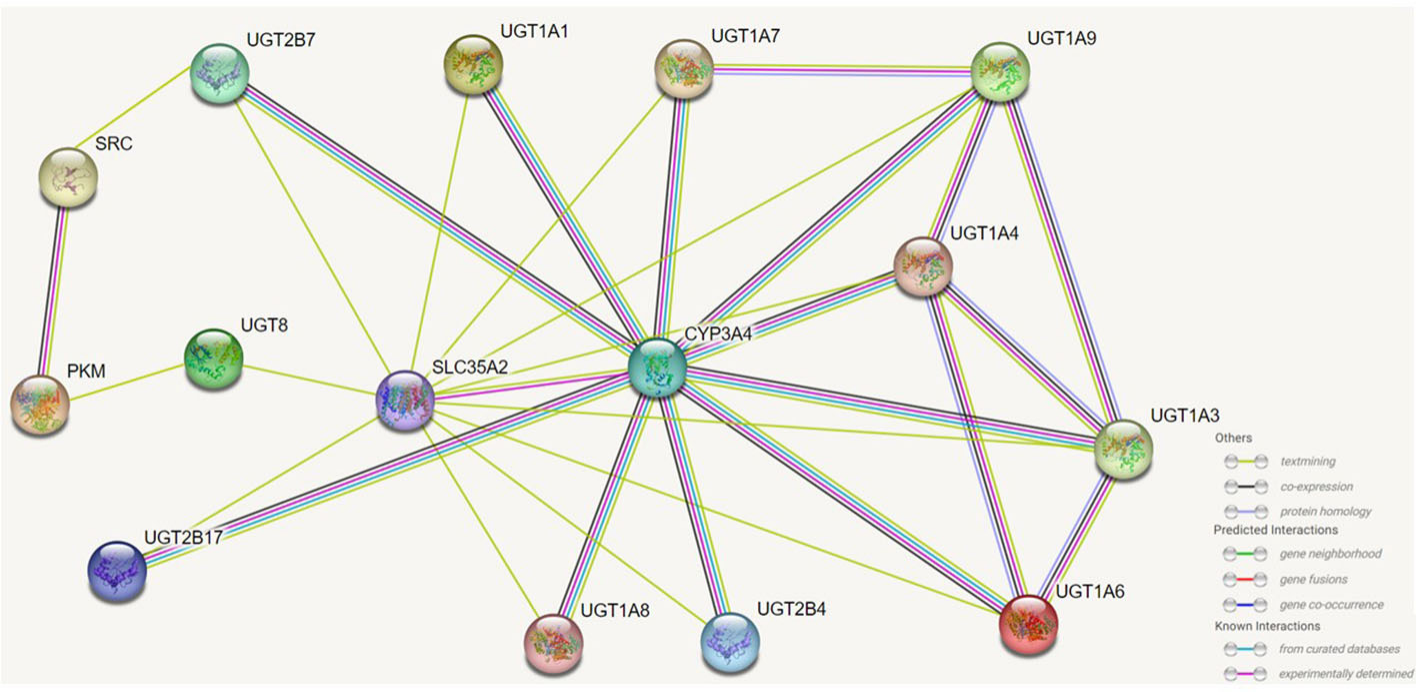
Effects of UGTs interacting with isotypic or heterotypic proteins on their function. UGTs can interact with proteins of the same isoform and different isoforms and the activity of UGTs will be altered and consequently the metabolism and capacity of the substrate will be altered. Through protein interactions, there are members of the UGTs family whose regioselectivity for substrates is also altered, and there are also interactions of UGTs with other proteins that can also localise the site of biochemical reactions. The figure lists which proteins UGTs can interact with, as well as predicting the way in which some of these proteins interact with each other.

When bound to UGT1A1, UGT1A1 and UGT1A7, CYP3A4 is able to promote the glucuronidation of these UGTs to their substrates and accelerate the rate of reaction between them (113). In prostate cancer, UGT2B17 can interact with the kinase c-Src, which is associated with the ability of c-Src to activate the receptors of various steroid hormones (114). UGT1A can generate its isoform protein UGT1A_i2s through alternative splicing, and in colon cancer cells UGT1A_i2s can interact with PKM2 to affect cellular energy levels, redox homeostasis and proliferative (115). And more importantly UGT1A_i2s can weaken the scavenging activity of catalase and peroxidase by interacting with them (116).UGT8 can interact in the endoplasmic reticulum with SLC35A2 to affect the balance between endoplasmic reticulum-localized lipid galactose and Golgi-localized protein galactose reactions. Furthermore, in the endoplasmic reticulum UGT8 also binds to the sigma-1 receptor (Sig-1R), and Sig-1R knockdown prolongs the lifespan and enhances the activity of UGT8 in the endoplasmic reticulum (23). UGT also interacts with microsomal proteins such as epoxide hydrolase 1, carboxylesterase 1, alcohol dehydrogenase and glutathione S-transferase, but the effects of these on the organism need further study (21–23) (**Figure 5**).

## 5 Inhibitors and inducers of UGTs

This review also focuses on the regulation of UGTs in cells. Thus, we want to explore whether there are clear drugs that can regulate UGTs. According to their effects on the expression and activity of UGTs, they were divided into two parts: inducers and inhibitors, and further clarified according to their drug types.

### 5.1 Inhibitors of UGTs

#### 5.1.1 Histone deacetylase inhibitors

Belinostat, a Histone deacetylase inhibitor, inhibits UGT1A1 in a dose-dependent manner, resulting in reduced elimination of SN-38, the active metabolite of irinotecan.Therefore, the efficacy can be enhanced in the small dose and will lead to severe drug toxicity at high doses (117).

#### 5.1.2 Tyrosine kinase inhibitors

It has been reported that many tyrosine kinase inhibitors have lower IC50 values than their clinical steady-state maximum concentrations in UGT1A1 inhibition assays *in vitro*, in contrast showing a higher incidence of hyperbilirubinemia *in vivo* experiments. It is concluded that UGT1A1-mediated inhibition of glucuronidation plays an important role (118).

#### 5.1.3 New uses for traditional medicines

Cao et al. found that zoledronic acid, a direct inhibitor of UGT8, effectively blocked the production of two downstream metabolites in the thiosemicarbazone biosynthetic pathway in a concentration-dependent manner, while significantly inhibiting the migration and invasion of breast cancer cells. These studies demonstrate that UGT8 is a potentially valuable target for tumor therapy (27, 119).

### 5.2 Inducers of UGTs

Studies have shown that estrogen upregulated UGT2B15 mRNA levels in a time-dependent and dose-dependent manner. Estrogen induced upregulation of UGT2B15 feedback regulate estrogen and androgen concentrations. As a consequence, estrogen regulate signaling in cancer cells and further exert regulatory functions (120) (**Table 4**).

**Table 4:** Summary of inhibitors and inducers of UGTs.

Name or type of the drugs	Object of action	Mode of action	Effect	Reference
Histone deacetylase inhibitor—Belinostat	UGT1A1	Inhibit UGT1A1 activity	Induce adverse drug reactions	(117)
Antiretroviral protease inhibitor—Atazanavir	UGT1A1	Inhibit UGT1A1 activity	Hyperbilirubinemia and Jaundice	(121)
Tyrosine kinase inhibitor—Nilotinib、Dabrafenib	UGT1A1、A7、A8、A9	Inhibit UGT1A activity	Increased risk of liver damage	(122, 123)
Chinese herbal—Ginseng saponin Rc	UGT1A9	Inhibit UGT1A9 activity	Not quite clear	(124)
Chinese herbal—Licochalcone A	Broad spectrum inhibition	Inhibit UGT1A、2B activity and expression	Induce adverse drug reactions	(125)
Zoledronic acid	UGT8	Inhibit formation of intermediate products	Inhibit the migration and invasion of breast cancer	(38, 119)
Estrogen	UGT2B15	Induce UGT2B15 expression	Regulate sex hormone concentrations and tumor signal transduction pathways	(120)
Neobavaisoflavone 、Isoflavone puerarin	UGT1A1	Induce UGT1A1 expression	Reduce therapeutic- related side effects	(126, 127)

## 6 Conclusions

The exploration of the connection between UGTs and cancer and the 22 recognized UGTs encoded by genes contained in the human genome provides basic information regarding the functional diversity of UGTs. The expression of UGTs is either high or low in different cancer types, and there are different prognostic manifestations in patients with different cancer types. The underlying mechanisms leading to the progression of such different cancers need to be studied and summarized in depth. Therefore, it is uncertain if UGTs have a positive or negative impact on cancer.

We can only hypothesize that an organism’s biological diversity may have various effects on cancer progression as more research shows that different UGTs in organisms alter their biological functions. For example, UGTs genes generate diverse protein variants through selective splicing. Gene splicing can be dynamically controlled since it is typically tissue-specific and can be quickly adjusted to the needs of cancer cell proliferation. Dysregulation of UGTs splicing may affect the regulation of tiny signaling molecules that increase the risk or progression of cancer because selective splicing dysregulation is a defining characteristic of cancer. Therefore, predicting the mechanisms affecting tumorigenesis during UGTs gene expression (including selective shearing, transcriptional, and post-transcriptional regulation) will provide new directions for early clinical tumor diagnosis and prevention.

In recent years, the investigation of tumor metabolism has focused heavily on the impact of UGTs on the pathways for glucose and lipid metabolism. We suggest that the mechanism may be related to UGTs and multiple non-UGTs protein interactions. The proteomics-identified UGTs-interacting proteins have an impact on glycolysis/glycogenesis and fatty acid breakdown. The observation that several UGTs conjugate various lipids, such as saturated fats, inflammatory cytokines, prostaglandins, citronellal, ceramides, diacylcyclohexanol, and triglycerides, is consistent with reports on the interaction of UGTs with lipid metabolism proteins, which suggest that they may have a broad role in basal lipid metabolism. As a component of the homeostatic system, UGTs may also control other pathways involved in the synthesis and utilization of UDP carbohydrates by protein–protein interactions. We presented how UGTs affect cancer metabolism based on the existing literature. If UGTs affect cancer development through other pathways is unknown to us.

## 7 Future perspectives

**Figure 6:**
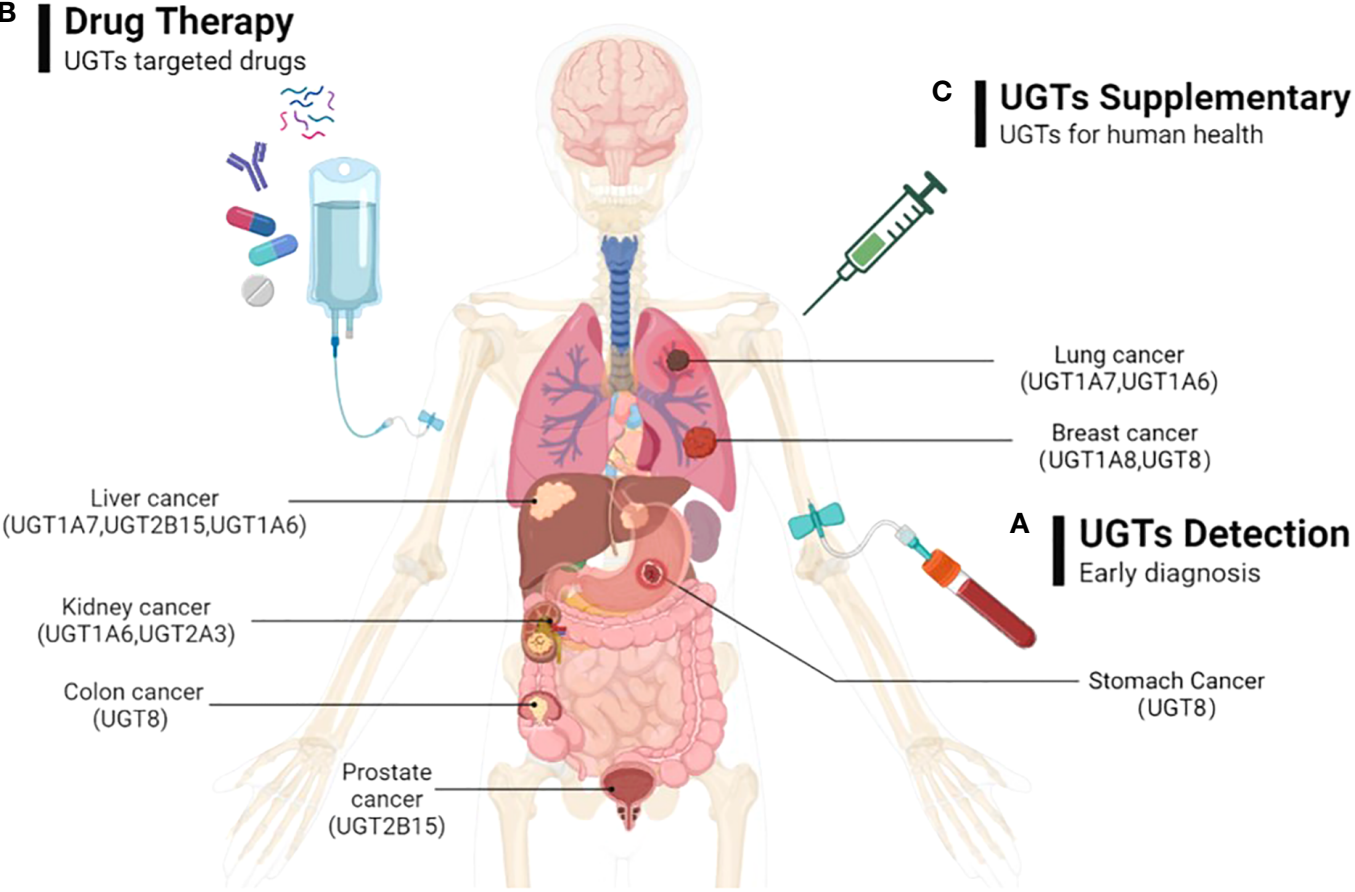
The possible directions of UGTs-related cancer therapy prospects. **(A)** By detecting the expression of UGTs to do early diagnosis of cancer; **(B)** Through the exploration of beneficial and safe UGT-targeted drugs with promising pharmaceutical applications to prevent and therapeutic UGTs-related cancers; **(C)** By injecting appropriate volumes of UGTs in cancer patients who were deficient in UGTs to supplement the deficient UGTs in the body.

As we mentioned above, the expression of UGTs varies among different cancers at different stages of progression. Abnormal UGTs expression undoubtedly affect the cellular response to endogenous or exogenous factors, and influence the cancer risk and progression of common malignancies, as well as their drug response. Therefore, early monitoring of expression of UGTs *in vivo* can provide a better understanding of the state of tumorigenesis and progression, which can provide new early diagnostic options for UGTs-related cancers. Such studies include monitoring UGTs mRNA and protein levels in different cancers and at different stages of cancer. And, the differences in UGTs expression in people in healthy and disease states should be considered. This would be a new means of early diagnosis of cancer (**Figure 6A**).

Currently, the development of selective UGTs inhibitors is in its infancy. Since endogenous and acquired drug glucuronidation is a new form of chemoresistance that is not easily overcome. Therefore, determining the increased or decreased expression of UGTs in specific cancers may help predict which class of drugs will experience glucuronidation. This will potentially help direct the selection of appropriate anti-cancer drugs. For cancer therapy, approaches that directly or indirectly target UGTs (e.g., UGT1A7, UGT1A8, UGT1A9, UGT8) may ultimately prove useful in slowing cancer progression, increasing drug-related responses, avoiding drug resistance, and ultimately improving patient prognosis. This would be a new cancer therapy option to consider (**Figure 6B**).

UGTs are highly expressed in tissues related to drug metabolism, promote drug metabolism *in vivo* through glucuronidation, and play a critical role in the metabolism of some antitumor drugs, thereby improving drug function and reducing drug toxicity. In some cancers, the deficiency of UGTs (e.g., UGT1A1, UGT1A8, UGT2B17, UGT1A1, UGT1A7) can lead to the development of tumor malignancy. Therefore, exogenous supplementation of UGTs is beneficial for cancer therapy. This would be a therapeutic modality that would help improve patient prognosis (**Figure 6C**).

## Author contributions

PH and YL designed this review. WL, JL and RZ drafted the manuscript. All the authors critically revised the manuscript and declare no conflict of interest. All authors contributed to the article and approved the submitted version.
